# A rare case of pemphigus vulgaris disguised as a malignant gingival ulcer

**DOI:** 10.1186/s12903-023-02980-6

**Published:** 2023-05-23

**Authors:** Jun Chen, Hui Tang, Ding Zhang, Yuqi Tang, Wenjie Li, Gui Liu, Binjie Liu

**Affiliations:** 1 Department of Periodontics and Oral Medicine, Xiangya Stomatological Hospital & Xiangya School of Stomatology, Hunan Key Laboratory of Oral Health Research & Hunan Clinical Research Center of Oral Major Diseases and Oral Health & Academician Workstation for Oral-maxillofacial and Regenerative Medicine, Central South University, Changsha, 410008 China; 2 Department of Orthodontics, Xiangya Stomatological Hospital & Xiangya School of Stomatology, Central South University, Changsha, 410008 China; 3 Department of Oral Pathology, Xiangya Stomatological Hospital & Xiangya School of Stomatology, Central South University, Changsha, 410008 China

**Keywords:** Pemphigus vulgaris (PV), Oral mucosa, Gingival ulcer

## Abstract

**Background:**

Pemphigus vulgaris (PV) is a kind of rare and severe autoimmune bullous disease. In this case, the specificity of oral PV lies in the clinical manifestations of a single palatal ulcer, and no blisters were found in the oral mucosa. This case provides a powerful reference for dentists diagnosing and treating oral PV with atypical clinical presentations.

**Case presentation:**

A 54 years old female patient presented with a non-healing palatal gingival ulcer for over three months. By histopathological H&E staining and the direct immunofluorescence (DIF) test, the final diagnosis was oral PV. After topical glucocorticoid therapy, the affected area was cured.

**Conclusions:**

In patients with prolonged erosion of the skin or oral mucosa, even if complete blisters are not visible, the physician should consider autoimmune bullous diseases and pay attention to avoid diagnostic defects.

## Background

Pemphigus vulgaris (PV) is a severe and chronic autoimmune bullous disease of the skin and mucosa [1]. The mucosal lesions were blisters and erosion. PV usually begins as multiple painful non-healing erosions of the oral mucosa that are followed by the development of flaccid vesicles and bullae, primarily involving the buccal mucosa, palate, gum, and other areas susceptible to friction [2]. The intraepidermal blisters of PV are flaccid and fragile, and Nikolsky’s sign was positive [1, 3]. In this case, the patient presented with a long-term, non-healing ulcer on the palatal gums of tooth ^#^27, with no noticeable blister. The specificity of this case is that no blisters were found on the gums or other parts of the oral mucosa. In addition, in the absence of epithelium, it was impossible to determine whether the vesicles were above the basement membrane or subepithelial, as the presence of inflammation is expected in an ulcer, making diagnosis difficult. We hope that this case will provide an essential reference for the clinical diagnosis and treatment of PV with atypical clinical manifestations.

## Case presentation

**Fig. 1:**
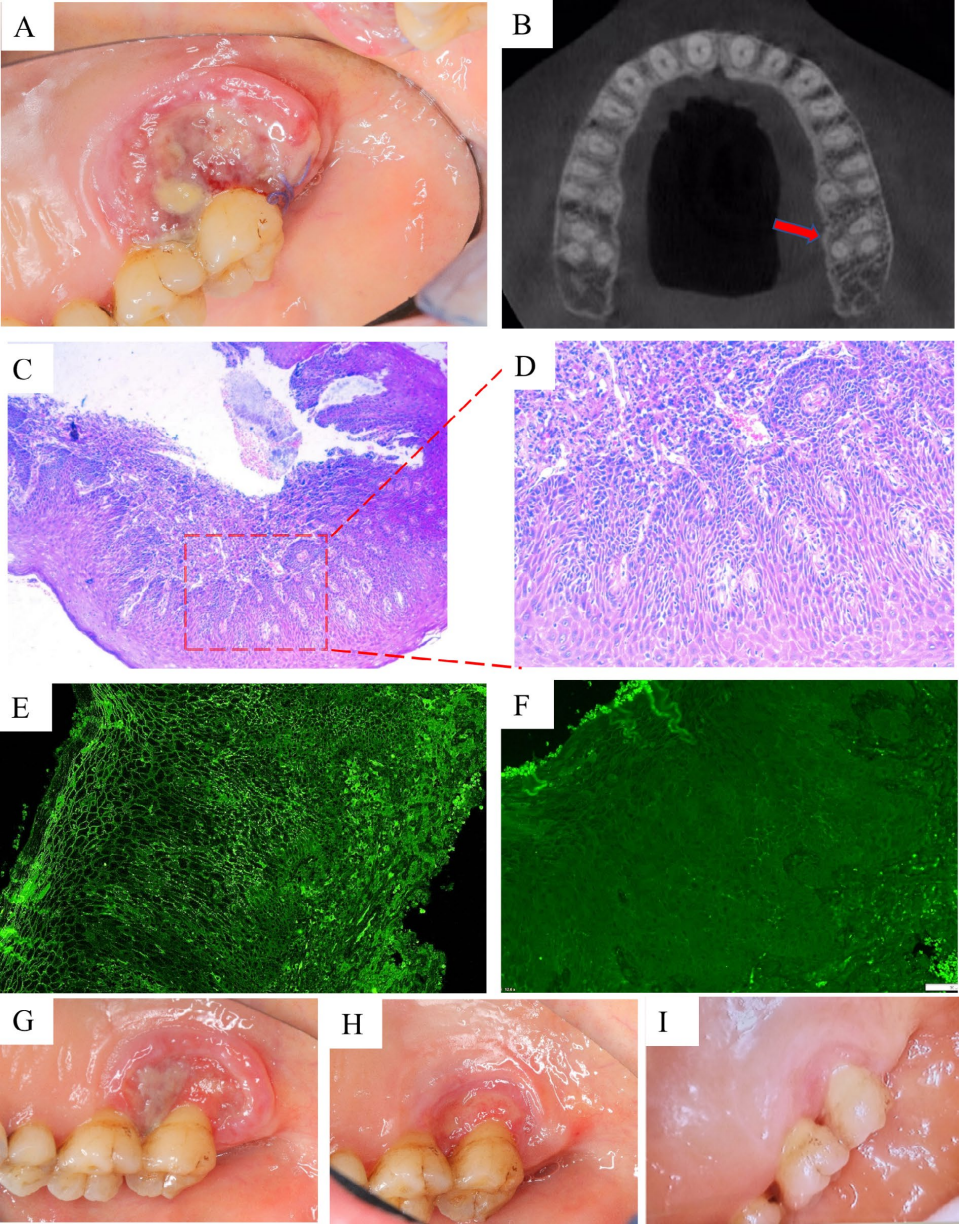
**(A)** An irregular ulcer-like lesion about 2*1.5 cm in size on the palatal gingival of tooth ^#^27. The lesion slightly protruded from the mucosal surface. **(B)** Cone beam CT (CBCT) showed that the buccal and palatal bone plates of tooth ^#^27 were continuous, without bone loss. **(C ~ D)** Histopathological image of the ulcer on the palatal gingival of tooth ^#^27 (H&E × 40). The pathological examination showed “chronic suppurative inflammation, ulceration with *Candida albicans* infection “(H&E × 100). **(E)** The direct immunofluorescence (DIF) assay showed positive IgG network deposition in epithelial and epidermal spinous cells. **(F)** The DIF assay showed that C3 is negative. **(G)** The image of the pemphigus vulgaris (PV) ulcer after 2 weeks of medication. **(H)** The image of the PV ulcer after 4 weeks of medication. **(I)** After 6 weeks of continuous local administration, the palatal PV ulcer was cured

A 54 years old female patient came to our Department of Oral Medicine due to a painful palatal gingival ulcer with difficulty chewing solid food on the left upper posterior teeth for over three months. She was administered some healing medication, but it did not work. She went to 4 different hospitals and was told it might be gingival cancer. Then, the patient was admitted to the Department of Oral and Maxillofacial Surgery, Xiangya Stomatological Hospital. Oral examination revealed an irregular ulcer-like lesion of the palatal gingiva on tooth ^#^27, about 2 × 1.5 cm in size. The lesion protruded slightly from the mucosal surface. The affected area is soft on palpation, easy to bleed, and with obvious pain (Fig. 1A). Oral hygiene was not poor, with calculus index (CI-S) = 0–2, debris index (DI-S) = 0–1, periodontal probing depth (PPD) = 2-4 mm, bleeding index (BI) = 1–2, and mild gingival edema. No blisters or ulcers were found in other parts of the mouth or skin. Cone beam CT (CBCT) showed that the buccal and palatal bone plates of tooth ^#^27 were continuous (Fig. 1B). The patient has no history of systemic diseases, allergies, bisphosphonate administration, radiotherapy, or chemotherapy. She also denied the recent use of immunosuppressants or the COVID-19 vaccine. The patient has no history of smoking, drinking, or chewing betel nuts.

By combing with clinical examination, course of disease, and history, the preliminary diagnosis was left maxillary gingival ulcer pending confirmation (Gingival cancer? Tuberculous gingival ulcer?). A tissue biopsy was performed. The pathological examination showed “chronic suppurative inflammation, ulceration with Candida albicans infection” (Fig. 1C ~ D). Then, she was referred to the Department of Periodontics and Oral Medicine, Xiangya Stomatological Hospital, for further diagnosis and treatment.

Generally, oral cancer, tuberculous ulcers, traumatic ulcers, bisphosphonate-related osteonecrosis of the jaw (BRONJ), radiotherapy, or chemotherapeutic oral mucositis are considered for oral ulcers that do not heal for more than two weeks [4–6]. In this case, the possibility of these five diseases was excluded by histopathological examination, CBCT, and medical history.

It has been reported that bullous diseases must be considered when oral ulcers do not heal for a long time [7]. However, in this case, the presence of intraepithelial or subepithelial blisters could not be determined by histopathology due to the severe inflammation and extensive infiltration of inflammatory cells. Therefore, a precise diagnosis could not be made directly through the histopathological results. The wax block from the original biopsy was deeply cut, and a direct immunofluorescence (DIF) assay was performed to aid the diagnosis. The results showed positive IgG, negative C3, and positive IgG network deposition in both epithelial and epidermal spinous cells, consistent with the diagnosis of oral PV [8] (Fig. 1E ~ F).

Once the diagnosis is precise, we start treating the patient. Since the patient has only one PV ulcer, we did not administer systemic hormonal medication. The patient was prescribed 0.12% chlorhexidine mouthwash three times a day. Then, triamcinolone acetate solution (5ml: 50 mg) was diluted five times with 0.9% sodium chloride solution and wet-dressed the affected area for 5–10 min, three times a day. After each wet dressing, gargle with 2.5% sodium bicarbonate solution for 5 min at 10-15-minute intervals. The patient was informed to review after every 2 weeks of medication. After 6 weeks of continuous local administration, the palatal PV ulcer was cured (Fig. 1G ~ I). At the 6-month follow-up, there was no evidence of recurrence.

### Discussion and conclusion

Pemphigus refers to a group of autoimmune bullous diseases of the skin and mucous membranes. PV is the most common and severe form of pemphigus [7]. In more than two-thirds of cases, PV begins in the oral mucosa and can last for several months. Thus, dentists play an essential role in early diagnosing and treating PV [9]. The duration of the bulla is ephemeral due to the fragile and easily ruptured PV blister wall, particularly in mucous membranes. Therefore, finding intact bullae in the oral cavity is rare and almost exceptional [7]. This explains why there were no blisters in the patient’s oral cavity in this case, but only a long-term non-healing ulcer was found on the palatal gingival of tooth ^#^27. In addition, in the absence of epithelium, it impossible to determine whether the vesicles were above the basement membrane or subepithelial, as the presence of inflammation is expected in an ulcer, leading to difficulties in diagnosis. In an extensive literature search, we found no evidence that inflammation could alter the histopathological outcome of PV. Further, DIF test showed positive IgG network deposition and negative C3, consistent with the diagnosis of oral PV [3, 9, 10].

In this case, the patient’s palatal gum of tooth ^#^27 developed a long-term, non-healing ulcer with no obvious blisters, which led most dentists to consider “gingival cancer” first. Fortunately, the final diagnosis was oral PV, but dentists need to be aware of the risk of oral PV ulcers becoming malignancy [11]. The particularity of oral PV in this case is that its clinical manifestation is a single palatal ulcer, rather than extensive erosion ulcer in the mouth, so it is deceptive. In an extensive literature search, we found no reports of PV with a single gingival ulcer of long-term non-healing as the chief clinical manifestation. Studies have shown that missed diagnosis and misdiagnosis are common in oral PV patients, even with a high frequency of oral involvement and easy access to the mouth [9]. This suggests the need to raise awareness and knowledge of the clinical oral manifestations of PV among dentists. This case provides a powerful reference for dentists diagnosing and treating oral PV with atypical clinical presentations. For any patient with skin or mucosal erosion, the physician should consider immune bullous disease to avoid diagnostic defects even if no complete blister is visible.

## Data Availability

We declared that materials described in the manuscript, including all relevant raw data, will be freely available to any scientist wishing to use them for non-commercial purposes, without breaching participant confidentiality. All data generated or analyzed during this study are included in this published article.

## Abbreviations

PV: Pemphigus vulgaris
DIF: Direct immunofluorescence
CI-S: Calculus index
DI-S: Debris index
PPD: Periodontal probing depth
BI: Bleeding index
CBCT: Cone beam CT
BRONJ: Bisphosphonate-related osteonecrosis of the jaw
